# Knowledge and attitude of pregnant women in Urmia, Iran, about oral health care during pregnancy

**DOI:** 10.1002/cre2.804

**Published:** 2023-11-03

**Authors:** Seyyed Amir Seyyedi, Afsoon Asadollahi, Zohreh Dalirsani, Zahra Abdollahzadegan, Mahla Rezaei

**Affiliations:** ^1^ Oral and Maxillofacial Medicine Department, Faculty of Dentistry Urmia University of Medical Sciences Urmia Iran; ^2^ Oral and Maxillofacial Diseases Research Center Mashhad University of Medical Sciences Mashhad Iran; ^3^ Faculty of Dentistry Urmia University of Medical Sciences Urmia Iran; ^4^ Department of Cosmetic and Restorative Dentistry, School of Dentistry Mashhad University of Medical Sciences Mashhad Iran

**Keywords:** attitude, knowledge, oral health, pregnancy

## Abstract

**Objectives:**

Physiological changes during pregnancy make mothers susceptible to periodontal diseases, in particular gingivitis, which could be prevented by good oral hygiene. Therefore, their knowledge and attitude could affect their oral hygiene and general health. This study therefore aimed to investigate the knowledge, attitude, and practice regarding oral hygiene, of pregnant women living in a city in Iran.

**Material and Methods:**

It was a cross‐sectional study that was performed in Urmia City in 2019. After completing an informed consent form, pregnant women completed a specially designed questionnaire, which included questions on demographic characteristics and assessed participants' knowledge and attitude about oral health care during pregnancy. A clinical examination of each participant's oral cavity was performed and dental plaque index (PI), gingival index (GI), and number of decayed, missed, filled teeth (DMFT) index were recorded. Any correlations between participants' knowledge and attitude and oral health indices were evaluated. A paired *t* test and Pearson's correlation coefficient were employed for statistical analysis.

**Results:**

A total of 96 pregnant women (mean age of 29.11 ± 6.80 years) participated in this study. Among them, 67 had a moderate level of knowledge. There was no significant correlation between participants' knowledge, and attitude levels and educational level (*p* = .88 and *p* = .43, respectively). Also, there was no correlation between knowledge and attitude levels and GI, PI, and DMFT (*p* > .05).

**Conclusions:**

This study showed that the knowledge and attitude of the pregnant women who participated were not favorable and their oral hygiene needed to be improved.

## “WHY THIS PAPER IS IMPORTANT” / “WHAT S KNOWN” / “WHAT S NEW”

- The pregnant women are susceptible to some oral diseases.
- Poor oral hygiene during pregnancy can affect the systemic health of mother and her fetus.
- Proper oral hygiene was not observed among pregnant participants.
- Knowledge and attitude of the pregnant women were not satisfactory and performance of oral health care needed to be improved.

## 1 INTRODUCTION

Pregnancy is a physiological condition with various changes in the oral cavity and other parts of the body, including, the respiratory, vascular, and hematologic systems and endocrine glands as well as some changes in eating behaviors. The increase in sex hormones, especially estrogen and progesterone predisposes pregnant women to the development of oral infections and some periodontal diseases, such as gingivitis, gingival hyperplasia, and pyogenic granuloma (George et al., 2012). This condition is caused by the intensification of the inflammatory response to local stimuli in response to the increase in the level of hormones and insufficient attention to oral hygiene due to mood swings (Naccasha et al., 2001). Poor oral health during pregnancy can affect the systemic health of both the mother and her fetus. It has been demonstrated that periodontal diseases increase the risk of adverse outcomes and complications of the pregnancy, such as preterm delivery, low‐weight birth, and pre‐eclampsia (Offenbacher et al., 2006). Maternal periodontal diseases indirectly lead to the birth of a premature infant with low weight through the transfer of bacterial products such as endotoxin and increased activity of inflammatory mediators (Mitchell et al., 2017). If a mother has periodontal problems, professional prophylaxis, and other periodontal treatment can be provided at any time during pregnancy. If a pyogenic granuloma lesion (or pregnancy tumor) has developed, the elimination of stimulating factors is recommended to reduce the size of the lesion (Caufield, 1997).

In addition, changes in the quality and quantity of saliva, poor oral hygiene, morning sickness, gag reflex, unhealthy diet, and tendency to increase carbohydrate intake lead to a potential increase in caries in pregnant women. Furthermore, there is evidence that the transmission of cariogenic bacteria from mothers to their infants can lead to the development of dental caries in children. Therefore, children whose mothers have poor oral hygiene are more prone to oral health problems (Jessani et al., 2016).

An important principle in designing dental treatment for a pregnant patient is to maintain an optimal level of oral hygiene. The most fundamental issue for improving oral hygiene is plaque control, which minimizes the inflammatory response of gingival tissue to hormonal changes during pregnancy (Mitchell et al., 2017). Therefore, maintaining oral health and preventing oral diseases before, during, and after pregnancy are important aspects of the general health of the mother and her fetus. However, pregnant women often neglect this important aspect of health (George et al., 2013).

Appropriate knowledge, attitude, and better performance of oral hygiene in pregnant women can prevent oral problems during pregnancy. In this study, the knowledge, attitude, and performance of oral hygiene of pregnant women during pregnancy were assessed using a specially designed questionnaire and evaluation of oral hygiene indices.

## 2 MATERIALS AND METHODS

### 2.1 Study design

**Figure 1:**
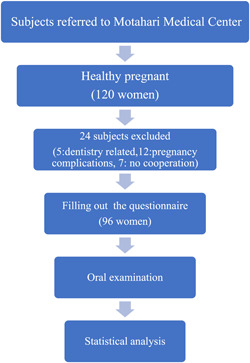
Flowchart for the selection of pregnant women.

This descriptive cross‐sectional study was performed in 2019. The subjects were recruited from pregnant women, attending the Motahari Medical Center, for regular visits. A total number of 120 pregnant women, who were referred to this clinic from March to July 2019, were asked to participate in this study. Of them, 96 who met the inclusion criteria were enrolled in the study (Figure 1).

### 2.2 Inclusion criteria

Good systemic health (absence of diseases affecting periodontium such as diabetes and blood coagulation disease); not taking the drugs affecting gingival health and causing abnormal bleeding such as phenytoin, nifedipine, cyclosporine, enoxaparin, corticosteroids, calcium channel blockers, warfarin, and antibiotics over the past 3 months; not smoking including cigarettes; lack of previously known oral disease; informed consent to participate in the study.

### 2.3 Exclusion criteria

Employment in professions related to dentistry; failure to complete the questionnaire completely; reluctance to participate in an oral examination; history of pregnancy complications, such as severe bleeding or pre‐eclampsia.

### 2.4 Data collection

First, all selected participants completed a three‐part questionnaire after signing the informed consent form. The first part of the questionnaire included demographic characteristics such as age, educational level, occupation, month of pregnancy, pregnancy characteristics, abortion history, number of previous pregnancies, and medical and medication history. The next two sections included questions that involved participants' knowledge and attitude and oral health practice during pregnancy.

This specially designed questionnaire was based on those used in previous studies and credible sources. The validity of the questionnaire was confirmed using the content validation method and after seeking the opinions of six oral medicine specialists. To verify the reliability of the questionnaire, it was evaluated by retesting. The questionnaire was completed for a second time by 15 pregnant women after an interval of 21 days. The correlation coefficient between the responses confirmed the reliability of the questionnaire (*r* = .86 for the attitude section and *r* = .75 for the knowledge section).

The knowledge section included 10 questions which were designed as three‐choice answers including “yes,” “no,” and “I do not know.” Each correct answer received a score of +1, every wrong answer a score of −1, and every neutral answer a score of zero. The highest score in this section was +10 and the lowest score was −10.

The attitude assessment section consisted of eight statements, which included four options based on the Likert scale: “I completely disagree,” “I disagree,” “I agree,” and “I completely agree.” The attitude section was scored between −16 and +16.

If the subjects were illiterate, in order not to bias the answers, the researcher would explain the questions to them, without changing her facial expression.

### 2.5 Oral examination

In the next step, an oral examination was performed by a specially trained dental student under the supervision of an oral medicine specialist, using a disposable dental probe and mirror.

The plaque index (PI) (O'Leary et al., 1972), the gingival index (GI) (Löe & Silness, 1963), and the number of decayed, missing, and filled teeth at the DMFT level, were recorded by the examiner. Because they were pregnant, caries were only assessed by clinical examination, and no dental radiographs were taken. Therefore, both enamel and dentine caries were recorded together. The number of decayed and filled teeth was separately recorded to determine whether the pregnant women had referred for necessary dental treatments prior to pregnancy. A DMFT score was then calculated.

The O'Leary et al. (1972) method was utilized for the evaluation of dental plaque. The participant first rinsed her mouth with water to remove any debris. Then, the subject was asked to use plaque‐disclosing tablets and rinse her mouth again. For each tooth, buccal, lingual, mesial, and distal surfaces were assessed. Finally, the number of colored surfaces compared to the total area was estimated as a percentage (O'Leary et al., 1972; Tibério et al., 2017).

The GI was assessed using the Löe and Silness method. The GI score of each surface of the tooth was defined as:

Normal gingiva scored as 0; mild inflammation: a slight change in color and edema without bleeding during probing scored as 1; moderate inflammation: existence of redness, edema, and bleeding during probing scored as 2; and severe inflammation: definite redness and edema, spontaneous wound, and bleeding scored as 3.

The score for each tooth was estimated by calculating the mean score of its four surfaces. According to the standard, if the GI of each tooth was from 0.1 to 1, it was classified as mild inflammation, from 1.1 to 2: defined as moderate inflammation, and from 2.1 to 3, considered as severe inflammation.

The resulting data were collected and analyzed using SPSS (version.18) software with paired *t* test and Pearson's correlation coefficient.

## 3 RESULTS

### 3.1 Characteristics of the participants

The results regarding the demographic characteristics of research subjects including age, gestational week, number of previous pregnancies, history of abortion, previous pregnancy problems, as well as dental problems in previous pregnancies can be seen in Table 1. The mean age of the pregnant women was 29.11 ± 6.80 years and the level of education in 56 (58%) of the subjects was below a high school diploma. Since pregnant women visited this health center in the morning, all of them were housewives.

**Table 1:** Characteristics of pregnant women in this study.

Variable	Minimum	Maximum	Mean ± SD
Age (years)	15	41	29.11 ± 6.80
Gestational week	3	38	15.57 ± 5.90
Number of pregnancies	0	7	2.3 ± 1.20
History of abortion	0	2	1.24 ± 0.42
History of previous pregnancy problems	0	2	1.02 ± 0.14
History of dental problems during previous pregnancies	0	2	1.25 ± 0.43

In terms of oral hygiene, out of the 96 participants, 83 (87%) reported brushing their teeth. Among them, 5 (5%) said they brushed three times a day, 11 (12%) twice a day, 62 (65%) reported brushing their teeth only once a day, and 5 (5%) rarely used a toothbrush. A few reported using dental floss or a mouthwash and eight did not use any product for oral care.

### 3.2 Knowledge and attitude

The mean participants' knowledge score was 2.31 ± 2.01, which was an indicator of a moderate knowledge level (Table 2).

**Table 2:** Levels of knowledge based on the educational level.

Educational level	Knowledge number (%)					
	Very good	Good	Moderate	Bad	Very bad	Total
Illiterate	0 (0%)	2 (25%)	5 (62.5%)	1 (12.5%)	0 (0%)	8 (100%)
Below high school diploma	0 (0%)	17 (30.4%)	38 (67.8%)	1 (1.8%)	0 (0%)	56 (100%)
High school diploma	0 (0%)	5 (23.8%)	15 (71.4%)	1 (4.8%)	0 (0%)	21 (100%)
Bachelor's	0 (0%)	2 (20%)	8 (80%)	0 (0%)	0 (0%)	10 (100%)
Master's	0 (0%)	0 (0%)	1 (100%)	0 (0%)	0 (0%)	1 (100%)
Mean ± SD	2.31 ± 2.01					

Regarding the attitude of pregnant women toward whether dental procedures are harmful to the fetus during pregnancy, 63 women (66%) agreed, 18 subjects (19%) disagreed, and the other 15 (15%) had no idea. It was found that 47 subjects (49%) had a neutral attitude toward dental treatment during pregnancy. The mean of pregnant women's attitudes score was 2.45 ± 2.21, which was an indicator of neutral attitudes toward oral health care during pregnancy (Table 3).

**Table 3:** Status of attitude based on the educational level.

Educational level	Attitude number (%)					
	Completely positive	Positive	Neutral	Negative	Completely negative	Total
Illiterate	1 (12.5%)	1 (12.5%)	5 (62.5%)	1 (12.5%)	0 (0%)	8 (100%)
Below high school diploma	2 (3.6%)	10 (17.9%)	26 (46.4%)	14 (25%)	4 (7.1%)	56 (100%)
High school diploma	1 (4.8%)	4 (19%)	9 (42.9%)	7 (33.3%)	0 (0%)	21 (100%)
Bachelor's	0 (0%)	1 (10%)	6 (60%)	3 (30%)	0 (0%)	10 (100%)
Master's	0 (0%)	0 (0%)	1 (100%)	0 (0%)	0 (0%)	1 (100%)
Mean ± SD	2.45 ± 2.21					

Moreover, 69 (72%) agreed that taking dental radiographs, even with a lead apron and thyroid collar, was potentially harmful to the fetus; 4 women (4%) disagreed and 23 (24%) had no idea about this issue.

### 3.3 Oral health indices

Three indices were used to evaluate the performance of pregnant women in oral hygiene. They were as follows: DMFT, GI, and PI. With regard to the DMFT Index, 52 participants (54%) had no filled teeth, indicating that they may not have visited a dentist for examination and treatment before their pregnancy. This could indicate poor knowledge of the necessity for dental examination and interventions before planning for pregnancy or that they had no dental caries and had no need to have any fillings.

The GI scores showed that 51 participants (53%) had mild inflammation, 42 (44%) had moderate gingivitis, and 2 (2%) had severe inflammation. The mean GI score was 0.98 ± 0.50 (Table 4).

**Table 4:** The levels of gingival index in pregnant women.

Gingival index (10+ to 10−)	Number (%)
Less than 0.1 (normal)	1 (1.04%)
0.1–1 (mild inflammation)	51 (53.12%)
1.1–2 (moderate inflammation)	42 (43.75%)
2.1–3 (severe inflammation)	2 (2.08%)
Total	96 (100%)
Mean ± SD	0.98 ± 0.50

The findings showed that the mean of dental PI among the participants was equal to 49.7 ± 22.1% with a minimum value of 2% and a maximum of 100%.

### 3.4 Multivariate analysis

Table 5 shows the correlation between women's performance including DMFT, GI, and PI with knowledge and attitude scores. Pearson's correlation coefficient revealed that there was no significant relationship between oral health indices with women's knowledge and attitude scores (*p* > .05). Also, there was no significant correlation between women's age, educational level, the number of previous pregnancies, and receiving oral health instructions before pregnancy with subjects' knowledge and attitude scores (*p* > .05) (Table 6).

**Table 5:** Correlation between DMFT, gingival index, and plaque index with knowledge and attitude scores.

Variables	Statistical tests	GI	PI	DMFT
Knowledge	Pearson correlation coefficient (*r*)	.102	.014	.107
	*p* value	.323	.895	.301
Attitude	Pearson correlation coefficient (*r*)	.033	−.044	.038
	*p* value	.784	.688	.713

Abbreviations: DMFT, decayed, missed, filled teeth; GI, gingival index; PI, plaque index.

**Table 6:** Correlation between age, education, number of pregnancies, and receiving oral instructions before pregnancy with knowledge and attitude scores.

Variables	Statistical tests	Age	Education	Number of pregnancies	Oral instructions before pregnancy
Knowledge	Pearson correlation coefficient (*r*)	−.017	.015	.077	−.048
	*p* value	.870	.888	.456	.642
Attitude	Pearson correlation coefficient (*r*)	−.014	−.081	.100	−.109
	*p* value	.894	.433	.333	.290

## 4 DISCUSSION

The results of this study indicated that the performance of oral hygiene in pregnant women referred to the Motahari Medical Center in 2019 was not optimal. Despite the special importance of oral health care during pregnancy, most pregnant women do not pay enough attention to oral hygiene.

However, these results were related to a government medical center, where pregnant women attended regular check‐ups in the morning. Therefore, it is expected that most of the participants in this study were housewives from low socioeconomic levels within the population.

Although more than half of the participants in the study had not obtained a high school diploma, the level of pregnant women's knowledge about oral health during pregnancy was not significantly related to their level of education. On the other hand, most mothers (67 women) had a moderate level of knowledge.

However, most dental treatments are not dangerous for a pregnant woman and her fetus, the results showed that nearly half of pregnant women (47 subjects) had a neutral attitude toward dental treatments during their pregnancy. Also, most pregnant women in the present study had a negative attitude toward taking radiographs during their pregnancy. Previous studies confirmed that it should be considered that high doses of X‐rays could be associated with low‐birth‐weight neonates. However, for most dental radiographs, the doses are below the risk threshold (Cohen‐Kerem et al., 2006; Ratnapalan et al., 2008).

In the present study, most pregnant women brushed their teeth only once a day. In a study of pregnant Lithuanian women, Vasiliauskiene (2003) found that only about 27% brushed their teeth twice a day, and more than half of them brushed only once a day. Moreover, the results of a study of pregnant Asian women living in the United Kingdom, found that they did not have good oral health practices during pregnancy, as 64.81% of them brushed only once a day, and 59% experienced bleeding during brushing (Ahmadian‐Yazdi & Sanatkhani, 2003). Also, 52.85% regularly visited the dentist at least once in 6 months and 22.22% reported that they visited the dentist just for relief of their pain or emergency treatments (Ahmadian‐Yazdi & Sanatkhani, 2003). In the current study, about 87% of women used a toothbrush, and most of them (65%) brushed once a day.

The results of the present study show that 53% of participants had mild gingivitis and 44% had moderate inflammation. Also, about 50% of women's dental surfaces were covered with dental plaque, indicating poor oral hygiene.

A previous study found that the performance of oral hygiene was good in only 34.4% of pregnant women (HajiKazemi et al., 2005). Christensen et al. (2003) reported that most pregnant women, who observed symptoms such as gingival bleeding during brushing, thought that their gingival condition was normal; therefore they did not improve their oral health practice (Christensen et al., 2003). These results indicate that pregnant women do not receive enough information about the importance of prenatal oral care, even during their dental examinations during pregnancy. The HajiKazemi et al. (2005) study also found that although pregnant women are somewhat aware of their oral health status, they avoid visiting the dentist due to incorrect knowledge. Therefore, using appropriate training can improve the oral health of pregnant women (HajiKazemi et al., 2005). Meanwhile, the level of knowledge and attitude of obstetricians and midwives plays an important role in encouraging pregnant women to improve their oral care.

In a study performed by Bahri et al. (2012) in the city of Mashhad, Iran, the levels of knowledge of pregnant women increased and their attitude and dental practices improved after an oral health education program.

In the current study, more than half of the pregnant women had a level of literacy below that of those with a high school diploma. The mean of DMFT in illiterate mothers was equal to 19.75 ± 5.97, and was higher than that of the literate mothers. However, there was not a significant correlation between educational level and DMFT status. It should be remembered that the present study was performed at a local government clinic, where most of the patients had a lower social status and lower levels of education. These factors might influence the results; therefore, these results could not be generalized for the whole population

On the other hand, since caries and filled teeth have the same value in a DMFT score, assessment of DMFT alone cannot be a good indicator for subjects' practice, because filling the teeth before the pregnancy, could be considered a positive indication of previous attendance at a dental clinic. It is suggested that each of the criteria of this index should be assessed separately and then compared with the knowledge and attitude scores and educational level.

Considering the results obtained regarding the status of knowledge, attitude, and performance of pregnant women with respect to their oral health and considering the necessity of oral health care in the pregnancy, it is recommended that another study on the effects of oral health education before pregnancy, on knowledge and attitude status and the performance of oral health care in pregnant women be performed. Furthermore, the correlation between oral health indices with consequences and complications of pregnancy should be evaluated.

Therefore, while trying to remove the structural barriers of the health system, including increasing insurance coverage and better access to healthcare centers, it is necessary to have appropriate policies for comprehensive training programs for society, especially reproductive‐aged women, in health centers. Normally, increased knowledge and an improved attitude are expected to affect health behaviors and gradually lead to better performance of oral hygiene. It is also recommended that oral health education be included in the educational curriculum of medicine, dentistry, and midwifery.

## 5 CONCLUSION

This study showed that the knowledge and attitude of its participating pregnant women were not satisfactory and their oral hygiene should be improved. It is generally expected that attitude and knowledge directly influence health behaviors. The findings indicate the necessity to provide educational programs targeting oral health care, especially for women of reproductive age.

## AUTHOR CONTRIBUTIONS

*Study conceptualization*: Seyyed Amir Seyyedi. *Data handling*: Zahra Abdollahzadegan. *Experimental design*: Seyyed Amir Seyyedi and Mahla Rezaei. *Analysis and interpretation of data*: Afsoon Asadollahi. *Provision of study materials and equipment*: Zahra Abdollahzadegan and Mahla Rezaei. *Study validation and data presentation*: Zahra Abdollahzadegan. *Supervision*: Seyyed Amir Seyyedi and Zohreh Dalirsani. *Draft preparation*: Zahra Abdollahzadegan and Seyyed Amir Seyyedi. *Study consultation*: Zohreh Dalirsani and Seyyed Amir Seyyedi. *Writing and reviewing and project administration*: Seyyed Amir Seyyedi and Zohreh Dalirsani. All authors approved the final version of this article.

## CONFLICT OF INTEREST STATEMENT

The authors declare no conflict of interest.

## ETHICS STATEMENT

The study process was carried out after the approval of the protocol of the study by the ethics committee of Urmia University of Medical Sciences (the ethical code: IR.UMSU.REC.1398.204). Pregnant women were enrolled in the study after signing an informed consent form. To keep the personal information of the participants and also to prevent bias, each of them was given a code. No therapeutic intervention with side effects was performed, and the examinations were completely safe and in accordance with the principles of infection control.

## Data Availability

The datasets generated during and/or analyzed during the current study are available from the corresponding author upon reasonable request.
